# Haplotype analysis from unmanned aerial vehicle imagery of rice MAGIC population for the trait dissection of biomass and plant architecture

**DOI:** 10.1093/jxb/eraa605

**Published:** 2020-12-26

**Authors:** Daisuke Ogawa, Toshihiro Sakamoto, Hiroshi Tsunematsu, Noriko Kanno, Yasunori Nonoue, Jun-ichi Yonemaru

**Affiliations:** 1 Institute of Crop Science, National Agricultural and Food Research Organization, Tsukuba, Japan; 2 Institute for Agro-Environmental Sciences, National Agriculture and Food Research Organization, Tsukuba, Japan; 3 University of Essex, UK

**Keywords:** Biomass, GWAS, haplotype, high-throughput phenotyping, MAGIC, QTL, rice, unmanned aerial vehicle (UAV), vegetation fraction

## Abstract

Unmanned aerial vehicles (UAVs) are popular tools for high-throughput phenotyping of crops in the field. However, their use for evaluation of individual lines is limited in crop breeding because research on what the UAV image data represent is still developing. Here, we investigated the connection between shoot biomass of rice plants and the vegetation fraction (VF) estimated from high-resolution orthomosaic images taken by a UAV 10 m above a field during the vegetative stage. Haplotype-based genome-wide association studies of multi-parental advanced generation inter-cross (MAGIC) lines revealed four quantitative trait loci (QTLs) for VF. VF was correlated with shoot biomass, but the haplotype effect on VF was better correlated with that on shoot biomass at these QTLs. Further genetic characterization revealed the relationships between these QTLs and plant spreading habit, final shoot biomass and panicle weight. Thus, genetic analysis using high-throughput phenotyping data derived from low-altitude, high-resolution UAV images during early stages of rice growing in the field provides insights into plant growth, architecture, final biomass, and yield.

## Teaser

Unmanned aerial vehicle imagery of a rice MAGIC population at the vegetative stage in a haplotype-based genetic study reveals novel insights into field biomass and plant architecture.

## Introduction

Crop breeding needs both molecular and phenotypic markers for plant biomass and yield. Molecular DNA markers are used to distinguish DNA polymorphisms tightly linked to agronomic traits such as disease resistance, abiotic stress tolerance, days to heading and plant architecture (Jena and Mackill, 2008; Moose and Mumm, 2008; Miura *et al.*, 2011; Hori *et al.*, 2016). DNA sequencing technology now allows massive DNA sequence data to be obtained, making it easy to find DNA markers and to genotype thousands of lines, even in crops with complex genomes. In searching for DNA markers linked to highly heritable and agriculturally important phenotypes, breeders generally perform genetic analysis using populations such as biparental recombinant inbred lines (RILs). They need to collect phenotype data from hundreds of lines over several years, because biomass and yield vary with environmental conditions. In addition, phenotypic markers associated with crop production remain elusive. Therefore, phenotyping is a bottleneck in breeding studies.

To overcome the bottleneck, various high-throughput phenotyping systems have been developed (Zhao *et al.*, 2019; Yang *et al.*, 2020). In indoor systems, precise shoot, root, grain and seed phenotyping can be performed (Tardieu *et al.*, 2017). Depending on the phenotypes of interest, red–green–blue (RGB), thermal, multispectral, hyperspectral and x-ray sensors are used. Because the system is indoors, various environmental conditions can be tested, and transgenic plants can be easily dealt with. High-throughput field phenotyping systems, on the other hand, use wheeled vehicles, motorized gantries or unmanned aerial vehicles (UAVs; Yang *et al.*, 2017; Gracia-Romero *et al.*, 2019; Zhao *et al.*, 2019). The first two are limited by location, but UAVs can automatically take images of large areas.

UAVs may be effective for plant phenotyping, especially in crops grown in large fields, because the natural performance of different lines can be observed. Furthermore, a series of digital images from UAVs can be combined into orthomosaic images by reference to ground control points (Jin *et al.*, 2017; Weiss and Baret, 2017), enabling time-course monitoring. For high-throughput phenotyping, high-resolution imagery is not typically captured with UAVs because imaging at higher resolution not only requires many more images at low altitude and longer flight times, but also greater processing power and data storage capacity. Methods to capture images with UAVs change depending on phenotype, experimental conditions (field size and amount of time), equipment (UAV and sensor) and computer for data analysis. UAVs can be equipped with various cameras to investigate phenotypes such as vegetation fraction (VF), plant height, architecture (spreading habit), drought adaptability and disease severity (Condorelli *et al.*, 2018; Zhang *et al.*, 2018; Chen *et al.*, 2019; Hassan *et al.*, 2019; Marcial-Pablo *et al.*, 2019; Ogawa *et al.*, 2019; Wang *et al.*, 2019) in populations in which quantitative trait loci (QTLs) for phenotypes can be revealed through genetic studies. However, the use of UAVs for phenotyping crop lines is still limited in breeding because the low spatial resolution of high-altitude UAV imagery is often insufficient for evaluating plant growth and architecture, and for estimating biomass and yield of individual lines. This highlights the need to obtain high-resolution images of plants and to understand what the digital phenotype data represent in crop production.

VF, often represented as plant density, ground cover, canopy cover or crop emergence, has been investigated by UAV imagery (Torres-Sánchez *et al.*, 2014; Duan *et al.*, 2017; Jin *et al.*, 2017; Makanza *et al.*, 2018) in various situations such as winter survival of wheat (Chen *et al.*, 2019), early growth of maize under low phosphorus conditions (Gracia-Romero *et al.*, 2017), and uniformity of emergence in potato (Li *et al.*, 2019). These applications suggest the importance of VF as an aerial index in remote sensing and utility of UAVs to reveal it. However, the degree to which VF reflects actual shoot biomass and the genetic factors involved in VF are not yet determined.

To select lines with ideal phenotypes from populations, genetic diversity is crucial, because it gives rise to phenotypic diversity. For this purpose, multi-parental inter-mated lines known as multi-parental advanced generation inter-*c*ross (MAGIC) lines are useful. The first MAGIC population was developed in *Arabidopsis thaliana* (Kover *et al.*, 2009) from 19 accessions and was analysed at the level of the founder haplotype. MAGIC lines have since been developed in rice, maize, wheat, barley, sorghum, tomato, chickpea, faba bean and cotton (Huang *et al.*, 2015; Sallam and Martsch, 2015; Sannemann *et al.*, 2015; Islam *et al.*, 2016; Ongom and Ejeta, 2018). We produced the Japan-MAGIC (JAM) rice population from four *japonica* and four *indica* cultivars with high grain yield and biomass in Japan (Ogawa *et al.,* 2018a, b), with comparable genomic proportions of the eight founders in all lines. The JAM population has higher phenotypic diversity than a set of four biparental RILs produced from the same eight parents. Information on more than ten thousand single-nucleotide polymorphisms in the individual JAM lines has been already obtained and translated into haplotype information. The JAM lines are thus suitable for genome-wide association study (GWAS) and the evaluation of haplotype effects on phenotypes of interest. We used these lines for high-throughput phenotyping and subsequent genetic analysis.

The aim of this study was to reveal what VF represents at the vegetative stage. We developed a method to analyse VF in JAM lines from orthomosaic images made from low-altitude, high-resolution UAV images. The haplotype-based genetic approach showed that VF was related to shoot biomass and plant architecture, and that VF partially influenced the final shoot biomass and yield. These results shed light on the effectiveness and usefulness of VF in high-throughput phenotyping in the field for rice breeding.

## Materials and methods

### Cultivation of JAM lines and parental cultivars

We previously developed the JAM population from eight founders (Ogawa *et al.*, 2018b): ‘Akidawara’ (AK), ‘Bekogonomi’ (BE), ‘Tachiaoba’ (TC), ‘Mizuhochikara’ (MI; all *japonica*), ‘Suwon 258’ (SU), ‘Takanari’ (TK), ‘Hokuriku 193’ (HO) and ‘Ruriaoba’ (RU; all *indica*). By developing 10 lines from 100 eight-way recombinants, we produced 981 JAM lines. We characterized the JAM lines in 2016 and 2017, and omitted lines with very low fertility, late heading, seed shattering, and highly heterogenous distribution of fertility and days to heading, which could affect yield. We selected 165 JAM lines (F_7_ in 2018 and F_8_ in 2019) with consideration of kinship, so as to not use more than three lines from the same eight-way recombinants. The 165 JAM lines were grown without replication according to standard procedures at National Agriculture and Food Research Organization in Tsukuba, Japan, in 2018 and 2019. The eight parental cultivars were grown with three replicates and assessed only in 2019. Seeds soaked in water at 28 °C for 2 d were sown in trays filled with soil and incubated at 30 °C in the dark for 2 d. Seedlings were grown in a paddy field in Kannondai, Tsukuba, for around a month, and then 33 seedlings per line were transplanted (11 plants, 18 cm apart × 3 rows 30 cm apart, no replicates) into a nearby paddy field, and grown for five months.

### Image capture by unmanned aerial vehicle (UAV)

We monitored the field by UAVs on shoot sampling dates ±1 d (Tables 1, 2). In brief, a Phantom 4 Pro UAV (P4P; DJI, Shenzhen, China) flew automatically above the field. The flight pattern and image capture were controlled by DJI GS Pro software (DJI). The software and the camera used the following settings: capture mode, time interval; speed, 1.0 m s^-1^; altitude, 10 m; front overlap ratio, 80%; side overlap ratio, 79%; gimbal pitch angle, −90°; image size, 3:2 (5472 × 3648 pixels); image format, JPG; white balance, cloudy; aperture, auto; shutter, auto; exposure compensation value, −1. In terms of the altitude of the UAV flight, we tested which altitude was suitable to obtain images. The lowest altitude to fly the P4P automatically and stably in the field was 10 m. By comparing the images at an altitude of 10, 15, 20, and 25 m, the lowest altitude condition was the best for subsequent analysis (Supplementary Fig. S1). To set the focus, the P4P was manually raised to 10 m, the camera was focused automatically on a region of the canopy, and the focus mode was changed to manual. As ground control points (GCPs), we made painted black and white markers at eight points on paved surfaces surrounding the field and precisely measured the latitude, longitude, and altitude of each point with a TCRP1205 surveyor (Leica, Heerbrugg, Switzerland).

**Table 1.** Dates of UAV flights and shoot collections in 2019.

	Date of transplanting	Date of UAV flight	Date of collecting shoots
First	22 May	10 June (19DAT)	11 June (20DAT)
Second	22 May	17 June (26DAT)	18 June (27DAT)
Third	22 May	25 June (34DAT)	24 June (33DAT)
Fourth	22 May	1 July (40DAT)	1 July (40DAT)

DAT: days after transfer into the field

**Table 2.** Dates of UAV flights and shoot collections in 2018.

	Date of transplanting	Date of UAV flight	Date of collecting shoots
First	16 May	7 June (22DAT)	6 June (21DAT)
Second	16 May	12 June (27DAT)	13 June (28DAT)
Third	16 May	18 June (33DAT)	19 June (34DAT)
Fourth	16 May	24 June (39DAT)	25 June (40DAT)

### Generation of orthomosaic images

Using Agisoft PhotoScan Professional v. 1.4.3 software (Agisoft, St. Petersburg, Russia), we generated an orthomosaic from each set of aerial images using the following steps, as described previously (Ogawa *et al.*, 2019): (i) the photos were aligned (accuracy, high); (ii) the GCPs were inputted; (iii) the mesh was built (surface type, height field; source data, sparse cloud); (iv) a digital elevation model was built (DEM; source data, sparse cloud); (v) colours were calibrated (source data, model; no calibration of white balance); (vi) orthomosaic images were built (surface DEM; blending mode, mosaic). The orthomosaics were analysed in ENVI v. 5.5 remote sensing software (Harris Geospatial, Boulder, CO, USA). The map projection was converted to UTM zone 54N (WGS-84) with a 2 mm per pixel resolution. The converted image was rotated 66° clockwise to match the long-side direction of the field with the lateral direction of the final output image. Then the image was resized to a rectangle (28 000 × 14 000 pixels), to include the field and the eight markers, to minimize file size and thus, processing time.

### Quantification of vegetation fraction (VF)

**Fig. 1.**
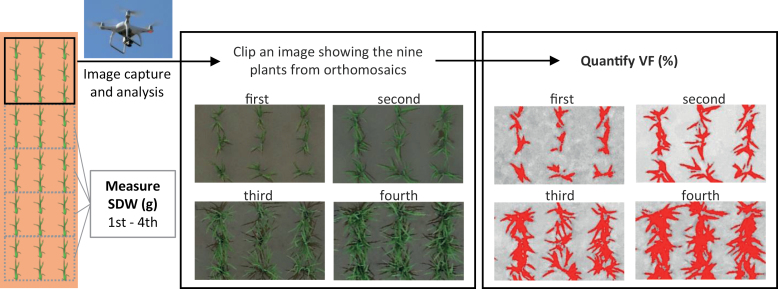
**Experimental design.** The 165 JAM lines were grown in 2018 and 2019 at Tsukuba, Japan, and analysed four times from around 20 to 40 d after transfer into the field, by using UAV images and shoot dry weight (SDW). RGB images with nine rice plants were converted to the L*a*b* colour space and vegetation fraction (VF) was calculated from the a* data. Six rice plants were collected from the field four times, and dried to measure SDW. The correlation between VF and SDW was examined.

Regions covering 3 × 3 plants of each JAM line and founders (450 × 270 pixels) in orthomosaic images were extracted for image analysis (Fig. 1). RGB data were converted to the L*a*b* colour space (León *et al.*, 2006) using ImageJ software (National Institutes of Health, USA). The a* data were used for auto-image thresholding by the Otsu method, to create binary images for extracting plant regions, and the percentage of the number of pixels of the plant region to the total number (450 × 270) of pixels was defined as the VF.

### Archery-target-style image analysis

**Fig. 2.**
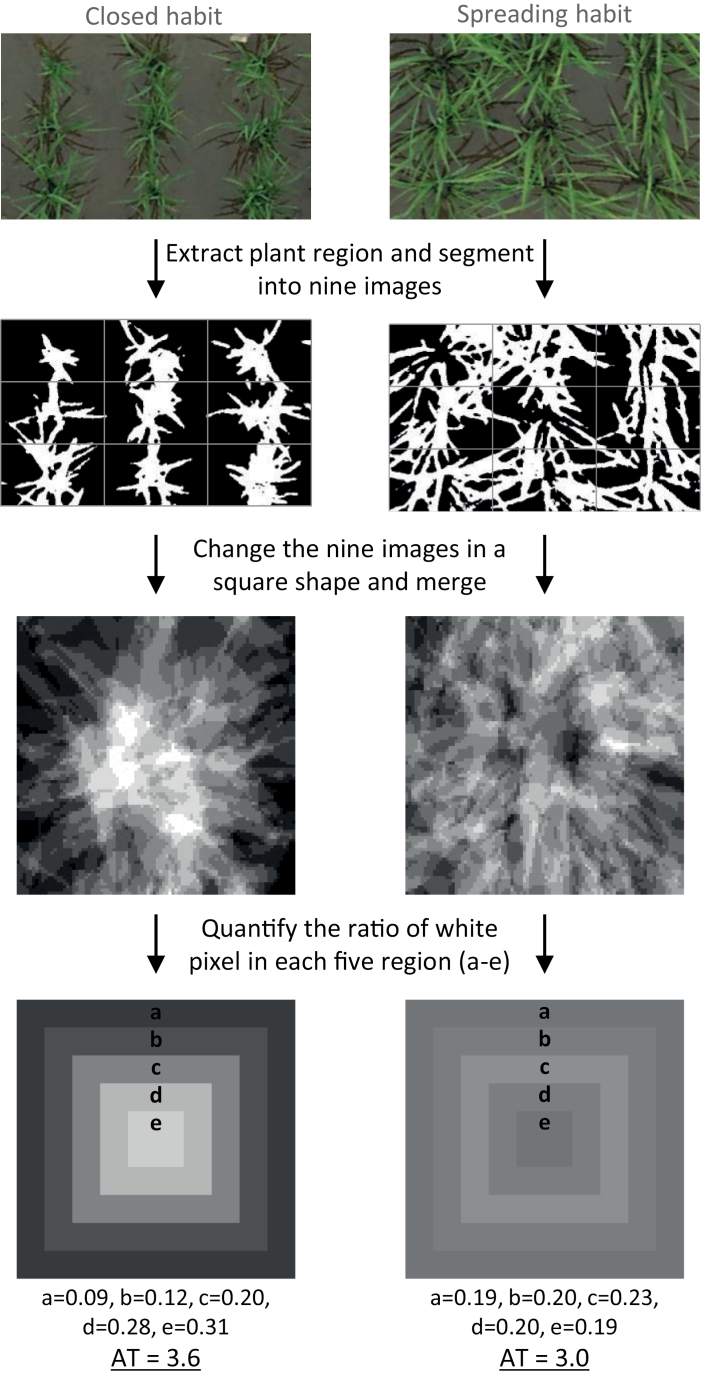
**Archery-target-style image analysis.** Use of image analysis to assess plant architecture from above. VF data of nine plants were merged. The ratio of the number of pixels of plant region to the total in regions ‘a’ to ‘e’ was calculated, weighted and summed to give AT values. Plant habit: (left) closed, (right) spreading.

The nine square binary images were extracted from the original VF image and merged on the basis of average pixel values (Fig. 2). The merged images were analysed by quantifying pixel ratios in each of five concentric regions, weighted from 1 to 5 from the outside to the centre, and summed as below. We named this method ‘archery-target (AT)-style image analysis’ and the obtained values as ‘AT values’. AT values were higher if plants were more upright.

$$\begin{array}{ll} AT= & \left(P_{a\_avg}\times 1\right)/P_{all\_avg}+\left(P_{b\_avg}\times 2\right)/P_{all\_avg}\\ & +\left(P_{c\_avg}\times 3\right)/P_{all\_avg}+\left(P_{d\_avg}\times 4\right)/P_{all\_avg}\\ & +\left(P_{e\_avg}\times 5\right)/P_{all\_avg} \end{array}$$

*P*_a_avg_ = average pixel value in region a (outermost)

*P*_b_avg_ = average pixel value in region b (1 in from outermost)

*P*_c_avg_ = average pixel value in region c (2 in from outermost)

*P*_d_avg_ = average pixel value in region d (3 in from outermost)

*P*_e_avg_ = average pixel value in region e (Innermost)

*P*_all_avg_ = average pixel value in all regions (a + b + c + d + e)

### Measurement of shoot dry weight (SDW)

We dug up six plants of each line on each of four dates in each year, which were in the same lane as those for VF analysis (Fig. 1). The plants were washed with water, and roots were removed with scissors to leave the shoots. The shoots were placed in a paper envelope and dried in a chamber (FJ-1100, Fujiwara Scientific Co. Ltd, Tokyo, Japan) at 80 °C for more than 2 d, and shoot dry weight (SDW) was recorded. SDW values are averages of six plants.

### Haplotype-based genome-wide association study (GWAS)

Haplotypes in the 165 JAM lines were estimated as in an Arabidopsis MAGIC population (Kover *et al.*, 2009), from sequencing data of the eight founders and of 13 603 SNPs, determined by genotyping-by-sequencing analysis. The haplotypes at each SNP were defined from the genotypes of the founders. Haplotype-based GWAS used haplotype information at each SNP, and phenotype data in non-parametric ANOVA (Kruskal–Wallis rank sum test) in the ‘kruskal.test’ package of R software. *P*-values obtained from the statistical analysis were used for the Manhattan plot. To identify QTLs for phenotypes, we detected peaks of the −log_10_*P* values in the GWAS results by using the ‘findpeaks’ function (minpeakdistance = 100) in the ‘pracma’ package of R software.

The effect of haplotype on phenotype at each QTL position was illustrated on scatter plots. If the number of haplotypes was 1 or 2, the data were discarded.

### Investigation of leaf sheath angle, panicle, stem and leaf weight

Leaf sheath angle (LSA) of plants is defined as the angle between leaf sheath and ground, and was measured with a laser range finder (GLM 50 C, Bosch, Gerlingen, Germany) at the vegetative stage (47 DAT; Ogawa *et al.*, 2019). LSA values of each JAM line were the averages of three plants. For measurement of panicle weight (PW) and stem and leaf weight (SLW), shoots of mature plants were dried for over a month in a drying room and cut 3 cm below the panicle base, to separate the parts. Total weight (TW) was calculated as PW + SLW. PW, SLW, and TW values of each JAM line are averages of five plants.

## Results

### Comparison of vegetation fraction with shoot dry weight

**Fig. 3.**
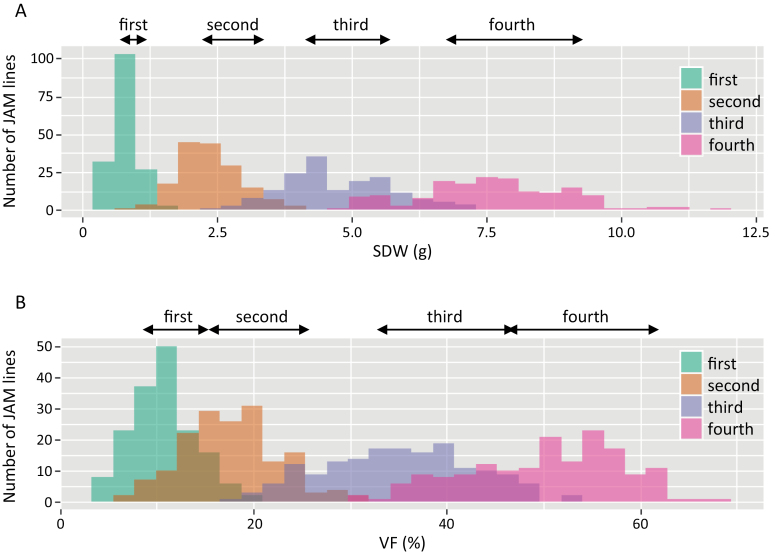
**Distribution of SDW and VF from first to fourth sampling dates in 2019. ↔** Range of phenotypes of the eight founders shown in Supplementary Figure S2.

To examine how the VF relates to shoot biomass, we compared UAV-derived VF with SDW on each sampling date. Over the 20 days from the first to the fourth sampling dates in each year, the mean SDW in the JAM lines increased to nearly 10× (Table 3). SDW on the fourth date ranged from 4.8 g to 11.9 g in 2019 and from 3.4 g to 15.0 g in 2018, indicating that shoot biomass differed widely in the JAM lines. The distribution of SDW on each date were roughly continuous and were wider in the JAM lines than in the eight founders (Fig. 3A, Supplementary Fig. S2).

**Table 3.** Summary of shoot dry weights (SDW) in the JAM lines in 2019 and 2018.

Year	Phenotype	mean (g DW plant^-1^)	SD	Min	Max
2019	SDW_first (20DAT)	0.78	0.23	0.39	1.71
2019	SDW_second (27DAT)	2.36	0.58	0.93	3.96
2019	SDW_third (33DAT)	4.74	1.01	2.43	7.16
2019	SDW_fourth (40DAT)	7.63	1.37	4.75	11.86
2018	SDW_first (21DAT)	0.76	0.20	0.32	1.47
2018	SDW_second (28DAT)	1.99	0.55	0.81	4.28
2018	SDW_third (34DAT)	3.76	0.96	1.73	7.01
2018	SDW_fourth (40DAT)	7.23	2.02	3.36	15.02

From the first to the fourth sampling dates, the mean of VF in the JAM lines calculated from the orthomosaics increased by 4.7× in 2019 and 3.9× in 2018 (Table 4). Similar to SDW, values varied beyond the range of the eight founders (Fig. 3B).

**Table 4.** Summary of VF in the JAM lines in 2019 and 2018.

Year	Phenotype	mean (%)	SD	Min	Max
2019	VF_first (19DAT)	10.8	3.2	3.9	19.5
2019	VF_second (26DAT)	17.8	4.9	7.0	31.4
2019	VF_third (34DAT)	35.0	7.7	17.4	54.0
2019	VF_fourth (40DAT)	50.1	8.0	30.3	67.8
2018	VF_first (22DAT)	10.5	3.8	4.0	22.1
2018	VF_second (27DAT)	20.1	6.8	7.4	40.4
2018	VF_third (33DAT)	29.2	9.0	12.6	55.1
2018	VF_fourth (39DAT)	40.9	9.1	17.3	63.5

**Fig. 4.**
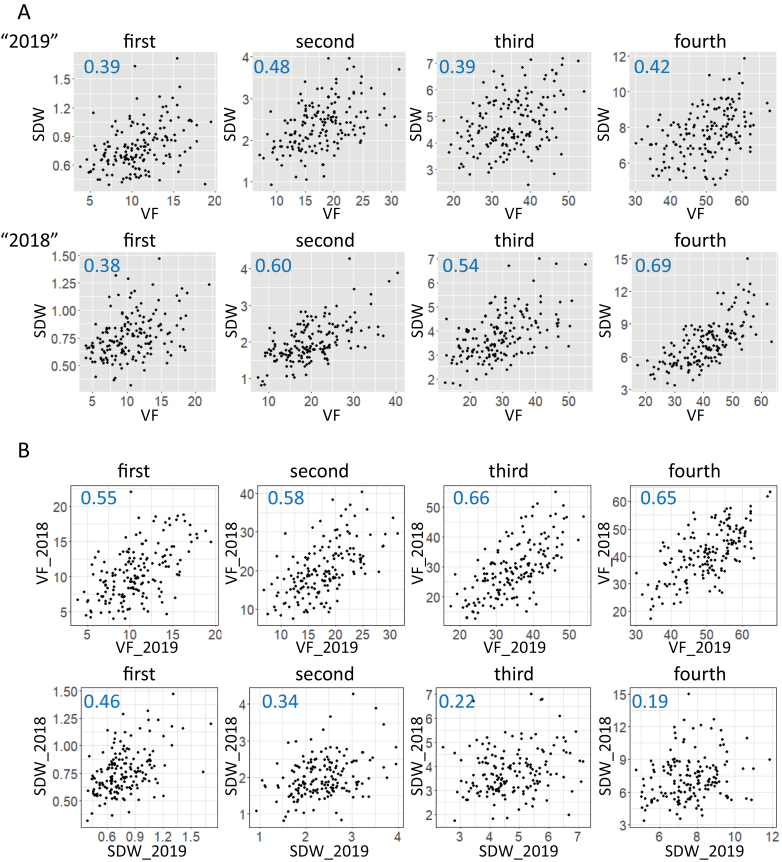
**Correlation between SDW and VF.** (A) Correlations between the two phenotypes in 2019 (upper row) and 2018 (lower row) in JAM lines. (B) Correlations of VF (upper row) and SDW (lower row) between years. Numbers in blue indicate Pearson’s *r*.

Pearson’s coefficient of correlation (*r*) between VF and SDW ranged from +0.4 to +0.7 (Fig. 4A, Supplementary Fig. S3) and fluctuated among dates. The correlation of VF between years were clearly higher than those of SDW (Fig. 4B, Supplementary Fig. S3). These results indicate that VF is a more robust phenotype than SDW, reflecting not only shoot biomass, but also other phenotypes, possibly including organ morphology and plant architecture.

### Detection of four QTLs for vegetation fraction by haplotype-based GWAS

**Fig. 5.**
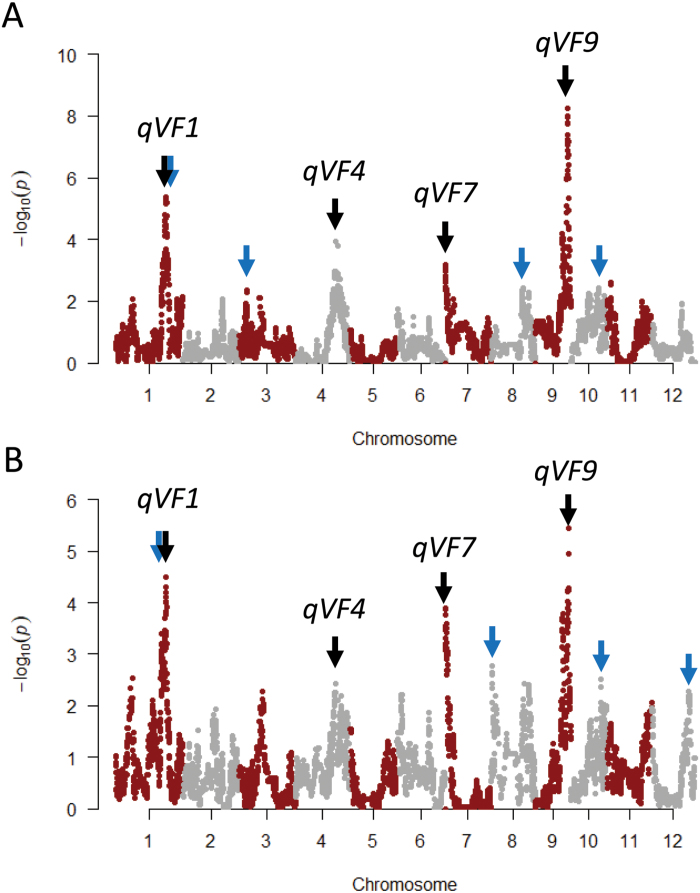
**GWAS of VF.** Manhattan plots of GWAS of VF on the fourth sampling date in (A) 2019 and (B) 2018 using haplotype data of 13 603 SNPs. Arrows indicate QTLs: black, common; blue, year-specific.

**Fig. 6.**
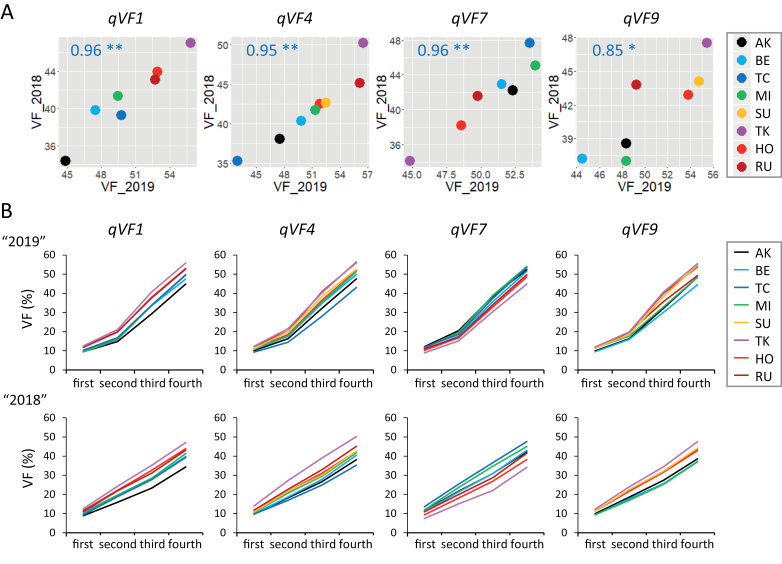
**Effect of haplotype on VF.** (A) Relationships of VF on the fourth date between 2019 and 2018. Average phenotypic values of the eight haplotypes at the four QTL positions are plotted. (B) Time-course pattern of haplotype effects on VF from first to fourth in 2019 (top) and 2018 (bottom).

To identify genetic loci involved in VF, we performed GWAS using VF data from each sampling date and haplotype data of the 13 603 SNPs (Supplementary Fig. S4). Among the eight strongest QTLs on the fourth date in 2019 and 2018, we found four common QTLs, named *qVF1*, *qVF4*, *qVF7* and *qVF9* (Fig. 5). The effect of haplotype at each QTL on VF was highly correlated between years (Fig. 6A). These QTLs were also detected on other dates (Supplementary Fig. S4), and the effect of the eight haplotypes on VF was relatively stable between years among the first to fourth dates (Fig. 6B). As ‘AK’, ‘BE’, ‘TC’ and ‘MI’ are *japonica* cultivars and ‘SU’, ‘TK’, ‘HO’ and ‘RU’ are *indica* cultivars (Ogawa *et al.*, 2018a,b), it seems that the *indica* haplotypes increase VF at *qVF1*, *qVF4* and *qVF9*, but that *japonica* haplotypes increase VF at *qVF7*.

**Fig. 7.**
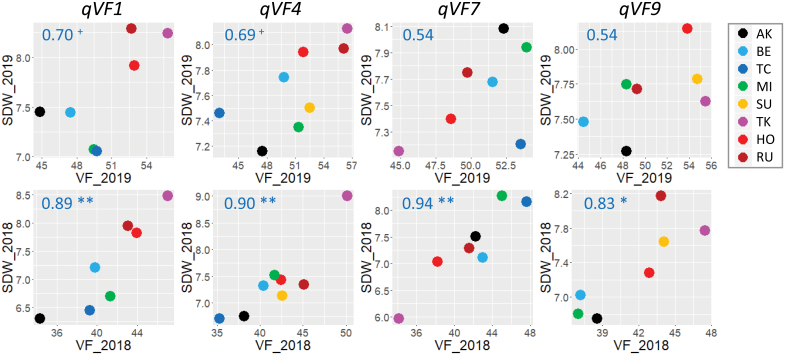
**Correlation of haplotype effect between SDW and VF at QTL.** Relationships between VF and SDW on the fourth date in 2019 (top) and 2018 (bottom). Numbers in blue indicate Pearson’s *r*. Asterisks indicate significant correlations (Pairwise two-sided, **P<0.01, *P<0.05, ^+^*P*<0.1).

To reveal whether the effect of the four QTLs on VF is correlated with shoot biomass, we plotted the effects of the eight haplotypes on VF and SDW at each QTL (Fig. 7). Correlations were significant at all four QTLs in 2018, but lower in 2019, and weak at *qVF1* and *qVF4*. Relatively reproducible correlations were detected at *qVF1, qVF7*, and *qVF9*, from the first to third dates (Supplementary Fig. S5). These results indicate that the QTLs for VF are also involved in shoot biomass, although the robustness depends on the QTL.

We also performed GWAS using SDW data (Supplementary Fig. S6). The plot patterns on the fourth date were largely changed between 2019 and 2018, except for *qVF1*, although *qVF7* and *qVF9* were detected a few times from the first to fourth dates in the two years. The time-course pattern of SDW effect (Supplementary Fig. S7) revealed a smaller difference among haplotypes than VF (Fig. 6B). The effect of the eight haplotypes on SDW at the four QTLs was often unstable among the first to fourth dates (Supplementary Fig. S7). These results indicate that SDW is an unstable phenotype even if data are recorded at the haplotype level, and that *qVF1* is associated most strongly with SDW.

### Relationship between VF and spreading habit

**Fig. 8.**
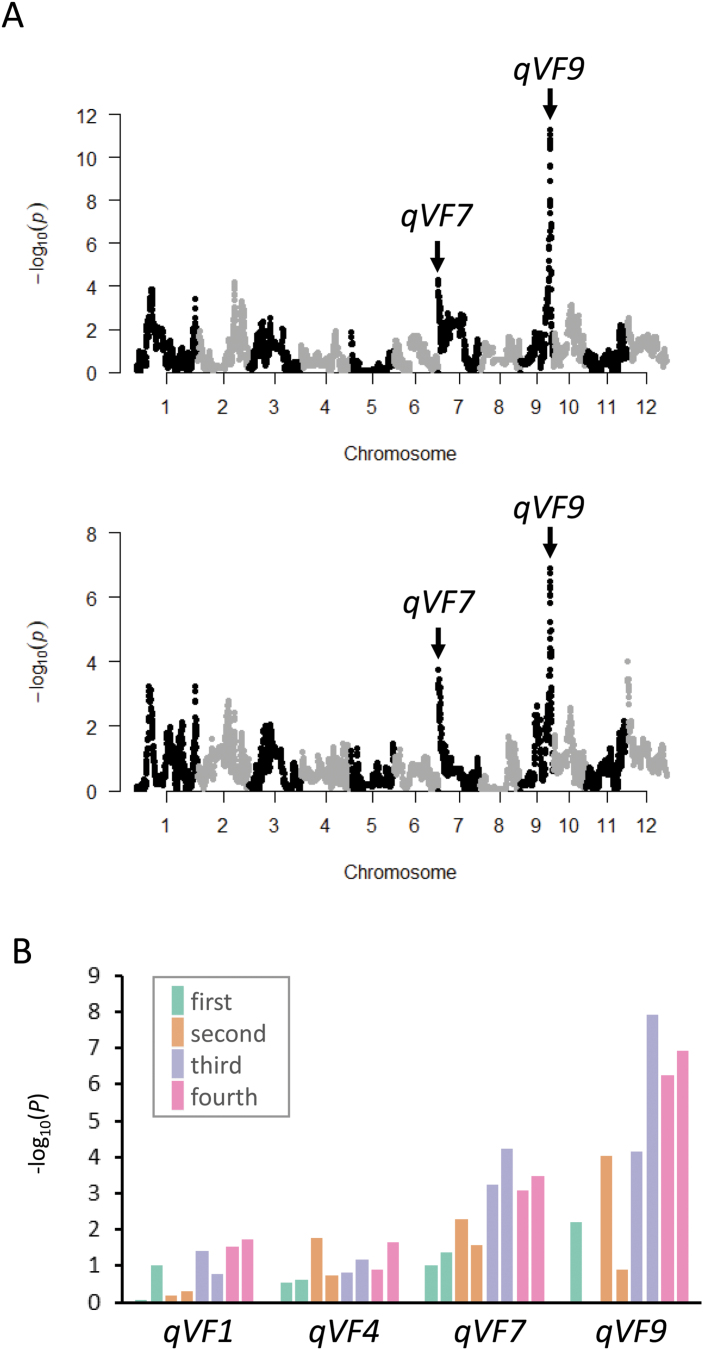
**Effect of plant architecture on VF at the four QTLs.** (A) Manhattan plots of haplotype-based GWAS of AT on the third date in 2019 (top) and 2018 (bottom). (B) *P* values in the GWAS at the four common QTLs. Bars of the same colour at each QTL indicate 2019 (left) and 2018 (right).

As described above, it is possible that VF is influenced by plant architecture, such as a spreading habit with inclined leaves and a compact habit with vertical leaves. To examine the possibility, we investigated the spreading pattern of the JAM lines at the QTL level using AT-style image analysis (Fig. 2). Haplotype-based GWAS of AT values in the JAM lines revealed clear peaks with low *P*-values at *qVF7* and *qVF9* (Fig. 8), implying that these QTLs affect the spreading or compact habit.

**Fig. 9.**
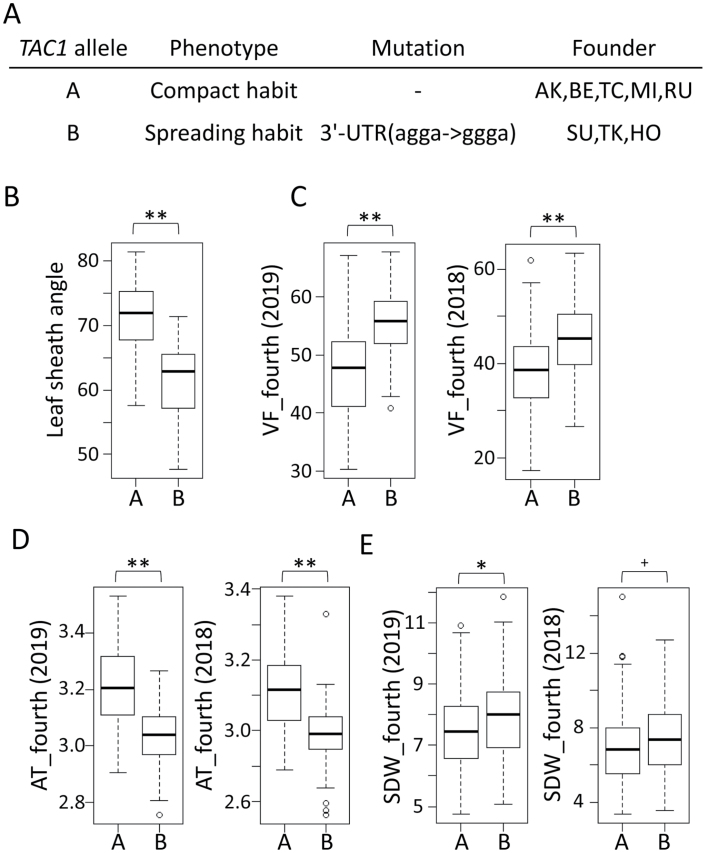
**Allelic effect of *TAC1* on VF and SDW.** (A) *TAC1* alleles of the eight founders. (B) Effects of *TAC1* alleles on leaf sheath angle. (C–E) Effects of *TAC1* alleles on (C) VF, (D) AT and (E) SDW on the fourth date. Asterisks indicate significant differences (Student’s *t*-test, **P<0.01, *P<0.05, ^+^*P*<0.1).

The SNP position of the top peak in *qVF9* is 20 402 463 on chromosome (Chr.) 9. This is near *Tiller Angle Control 1* (*TAC1*: Os09t0529300; 20 731 589–20 734 728 on Chr. 9), which is a major regulator of tiller angle (Yu *et al.*, 2007). The causative mutation is ‘AGGA’ to ‘GGGA’ in the 3’-UTR of *TAC1*. The mutation was found in ‘SU’, ‘TK’ and ‘HO’, but not the other founders (Fig. 9A). Furthermore, LSA was lower with those haplotypes at *TAC1* (Fig. 9B), indicating a spreading habit. VF was increased and AT was decreased in the JAM lines with those haplotypes at *TAC1* (Fig. 9C, D). These results suggest that *TAC1* is a candidate gene for *qVF9,* and that *qVF9* may affect VF by influencing the spreading habit. Notably, the ‘SU’, ‘TK’ and ‘HO’ haplotypes slightly increased SDW (Fig. 9E).

### Effect of VF on final shoot biomass and yield

**Fig. 10.**
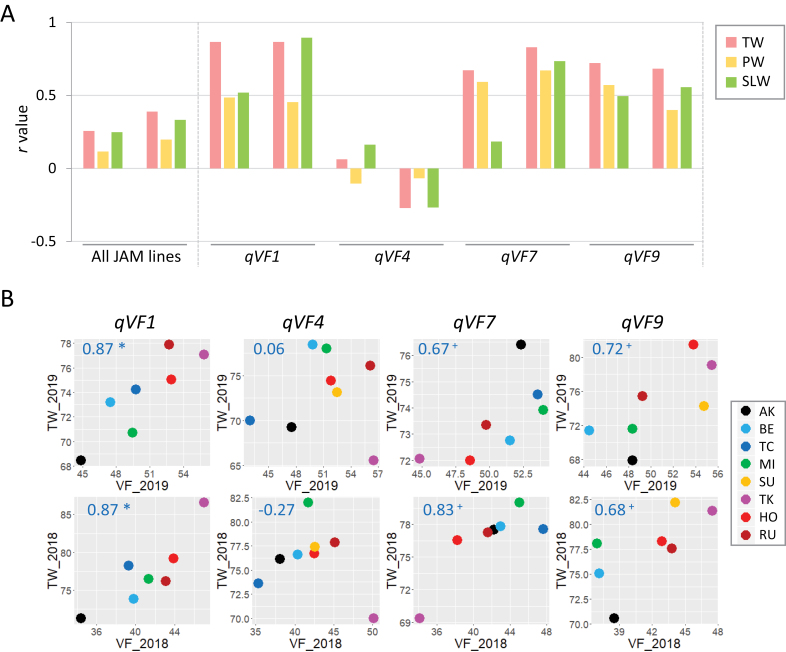
**Effects of the four QTLs for VF on final yield.** (A) Pearson’s *r* between VF on the fourth date and total weight (TW), panicle weight (PW), or stem and leaf weight (SLW) in 2019 (left trio of bars) and 2018 (right trio of bars) at the haplotype level. As a control, the correlations between VF and TW, PW and SLW data at the JAM line level are shown on the left. (B) Plots of the correlation between VF and TW in 2019 (top) and 2018 (bottom) at the haplotype level. Numbers in blue indicate Pearson’s *r*. Asterisks indicate significant correlations (Pairwise two-sided, *P<0.05, ^+^*P*<0.1).

We examined whether VF at the vegetative stage affects final shoot biomass and yield. We measured TW as total shoot biomass and PW as yield, in addition to SLW. We found a weak positive correlation between VF (fourth date) and TW in both years, but not PW (Fig. 10A, Supplementary Fig. S8). At the haplotype level, we found a correlation between VF and TW at *qVF1*, *qVF7* and *qVF9* (Fig. 10B, Supplementary Table S1). Interestingly, at *qVF7*, VF was correlated with TW and PW, but not with SLW (Fig. 10, Supplementary Fig. S9). These results indicate that VF at the vegetative stage partially affects final shoot biomass and yield in the JAM lines.

We also performed GWAS using TW, PW and SLW data (Supplementary Fig. S10). The *qVF9* was detected as a QTL for TW and SLW. In terms of the plot pattern of the GWAS, many peaks with low -log_10_ P values were found, majority of which were not common in the two years. This is similar to the tendency of plot patterns in GWAS using SDW at the vegetative stage (Supplementary Fig. S6), but not VF (Fig. 5, Supplementary Fig. S4). These results highlight the phenotypic stability of VF.

## Discussion

In this study, we showed that VF at the vegetative stage is correlated to shoot biomass in JAM lines. But the correlations were not very high, indicating that VF represents more than biomass. On this point, we believe that the haplotype approach worked well by identifying the correlations for the four common QTLs for VF. The result implies that VF contributes to shoot biomass at the QTL level. VF was a more robust phenotype than shoot biomass, and the four QTLs for VF were reproducible, which is suited to DNA marker–assisted breeding. Our results therefore show that VF is an effective and beneficial phenotype for high-throughput phenotyping in the field for breeding.

One weakness of orthomosaics derived from UAV images is the lack of side views, which provide information such as leaf length, tiller number and angle, which indoor high-throughput phenotyping systems can easily measure (Tardieu *et al.*, 2017; Wu *et al.*, 2019). AT-style image analysis of plant spreading pattern revealed the QTLs *qVF7* and *qVF9*, the latter of which could be *TAC1*, a gene for tiller angle. By providing insight into plant architecture, this analysis may partially overcome the weakness of UAV imagery. A method we developed to identify panicle position by detecting red flags attached to panicle bases can also overcome this weakness (Ogawa *et al.*, 2019).

We characterized four common QTLs for VF (Table 5). VF was increased in *indica* haplotypes at *qVF1*, *4* and *9* but in *japonica* haplotypes at *qVF7*. Among the eight haplotypes, ‘TK’ showed clear effects at the four QTLs. The effect of haplotype on VF was associated with that on SDW at all QTLs. Notably, *qVF1*, *7*, and *9* affected TW, and *qVF7* also affected PW. This indicates that vegetative growth partially affects final shoot biomass and yield, reinforcing the utility of UAV observation at the vegetative stage for breeding. Intriguingly, the involvement of *qVF7* and *qVF9* also in AT data of plant spreading pattern may imply that plant architecture directly affects final shoot biomass and yield.

**Table 5.** Summary of features of *qVF* QTLs.

QTL	Chr	SNP position ^a^	Haplotype with		Effect on				Candidate gene
			highest effect ^b^	lowest effect ^c^	SDW	TW	PW	AT	
*qVF1*	1	30 058 874	TK (*indica*)	AK (*japonica*)	+	+	–	–	–
*qVF4*	4	26 251 090	TK (*indica*)	TC (*japonica*)	+	–	–	–	–
*qVF7*	7	265 293	TC (*japonica*)	TK (*indica*)	+	+	+	+	–
*qVF9*	9	20 402 463	TK (*indica*)	BE (*japonica*)	+	+	–	+	*TAC1*

a: SNP position where the peak of −log_10_*P* values in haplotype-based GWAS in 2019 selected by ‘findpeaks’ package in R software.

b: Haplotypes showing the highest effect on VF.

c: Haplotypes showing the lowest effect on VF.

QTL: quantitative trait locus

Chr: chromosome

SNP: single-nucleotide polymorphism

SDW: shoot dry weight

TW: total weight (panicle weight + stem and leaf weight)

PW: panicle weight

AT: value obtained from ‘archery-target-style image analysis’

TK: ‘Takanari’

AK: ‘Akidawara’

TC: ‘Tachiaoba’

BE: ‘Bekogonomi’

*TAC1*: *Tiller Angle Control 1*

Our genetic analysis revealed that the candidate gene for *qVF9* is *TAC1*, which determines the degree of plant spreading or closed habit (Yu *et al.*, 2007). At *qVF1*, *qVF4* and *qVF7*, the haplotype effect on VF differed between *indica* and *japonica* founders. Many genes differ between *indica* and *japonica*, so haplotype-based allele mining (Ogawa *et al.*, 2018b) cannot narrow down candidate genes for the QTL. Searching for candidate genes requires further forward-genetic studies, including the production of near-isogenic lines. This will help to explain how the QTLs contribute to VF, biomass, plant architecture, and yield.

By contrast, it is desirable that lines with high biomass and yield can be selected from the population using digital data from UAV without genomic information. But the prediction of biomass and yield using only VF is likely to be hard, because the correlation between VF and SDW varied widely (*r* = 0.4–0.7). This may reflect environmental conditions. In fact, we also found year-specific QTLs for VF, which may be responsible for this variation. Further investigation of VF after the fourth sampling date would not be meaningful because the canopy would become more closed, so differences in VF between lines would become small. For the accurate prediction of final biomass and yield, modelling using more phenotypic data such as normalized difference vegetation index, biotic/abiotic stress tolerance, plant architecture, and environmental data such as weather and soil, should be attempted.

This study shows the phenotypic significance of the use of data collected by low-altitude, high-resolution UAV imagery for the evaluation of crop lines during the vegetative stage in the field. It is noteworthy that VF data were obtained easily and non-invasively in the field by using a commercial UAV equipped with a low-cost RGB camera and high-powered inexpensive computers for image analysis, which will be desirable for achieving agricultural application. Furthermore, this work reaffirms the usefulness of the haplotype-based genetic approach. As far as we know, this is the first case of the introduction of digital phenotypes into a haplotype approach using a MAGIC population. By selecting haplotypes with the highest effect on VF using DNA marker–assisted selection at the four QTLs, we should be able to select lines with the highest VF values, which are expected to be high-yielding. This study shows a model case toward massive digital data–driven breeding using haplotype information and high-throughput phenotyping in the field.

## Supplementary data

The following supplementary data are available at *JXB* online.

Fig. S1. Difference in image resolution by UAV flight altitude.

Fig. S2. Shoot dry weight and vegetation fraction of JAM’s parental lines in 2019.

Fig. S3. Pearson correlation between all shoot dry weights and vegetation fractions in two years’ experiments using the JAM lines.

Fig. S4. Manhattan plot of GWAS regarding vegetation fraction of JAM lines in 2019 and 2018.

Fig. S5. Average phenotypic values of the eight haplotypes at the four QTL positions.

Fig. S6. Manhattan plot of GWAS regarding shoot dry weight of JAM lines in 2019 and 2018.

Fig. S7. Time-course pattern of the effect of eight haplotypes on shoot dry weight.

Fig. S8. Scatter plots of vegetation fraction (fourth) and yield traits.

Fig. S9. Effects of the four QTLs for vegetation fractions on panicle weight.

Fig. S10. Haplotype-based GWAS regarding total weight, panicle weight, and stem and leaf weight.

Table S1. Haplotype effect on total weight, panicle weight, and stem and leaf weight at the QTLs for vegetation fractions.

eraa605_suppl_Supplementary_Figure

eraa605_suppl_Supplementary_Table

## Data Availability

The data supporting the findings of this study are available from the corresponding author (DO), upon request.

## Abbreviations

Chr: chromosome
CL: culm length
DAT: days after transfer into the field
AT: archery-target
DEM: digital elevation model
GCP: ground control point
GWAS: genome-wide association study
LSA: leaf sheath angle
MAGIC: multi-parental advanced generation inter-cross
PW: panicle weight
QTL: quantitative trait locus
RILs: recombinant inbred lines
RGB: red-green-blue
SDW: shoot dry weight
SLW: stem and leaf weight
TW: total weight
UAV: unmanned aerial vehicle
VF: vegetation fraction
