# Supplementary material for: Haplotype analysis from unmanned aerial vehicle imagery of rice MAGIC population for the trait dissection of biomass and plant architecture
Source: J Exp Bot. 2020 Dec 26;72(7):2371–82. doi: 10.1093/jxb/eraa605 (PMC8006554; doi:10.1093/jxb/eraa605)
Supplement: eraa605_suppl_Supplementary_Figure [file eraa605_suppl_supplementary_figure.pdf]

Haplotype analysis of data from unmanned aerial vehicle imagery of rice MAGIC population for trait dissection of biomass and plant architecture

Daisuke Ogawa[a1]*, Toshihiro Sakamoto[b1], Hiroshi Tsunematsu[a], Noriko Kanno[a], Yasunori Nonoue[a], Jun-ichi Yonemaru[a]*

[a] Institute of Crop Science, National Agricultural and Food Research Organization, Tsukuba, Japan
[b] Institute for Agro-Environmental Sciences, National Agriculture and Food Research Organization, Tsukuba, Japan

Supplementary Fig. S1-S10

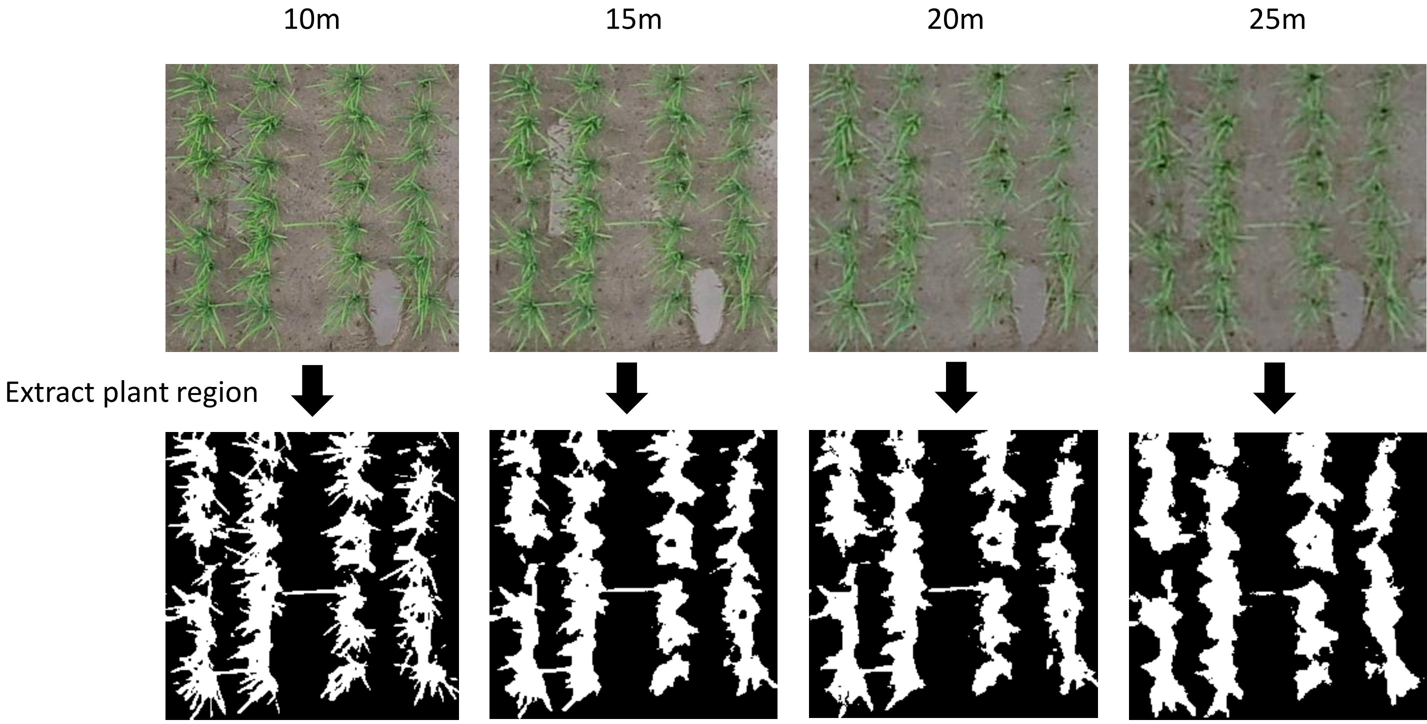

Supplementary Figure S1. Difference in image resolution by UAV flight altitude. To examine how flight altitude affects extraction of plant region, we took pictures of rice plants in the paddy field at an altitude of 10, 15, 20 and 25 m using Phantom 4 Pro UAV. The raw RGB data were converted to the L*a*b* colour space. As a result of auto-image thresholding by the Otsu method to create binary images for extracting plant regions using a* data, the outline of the plant in the binary image was most distinct when taken from an altitude of 10 m.

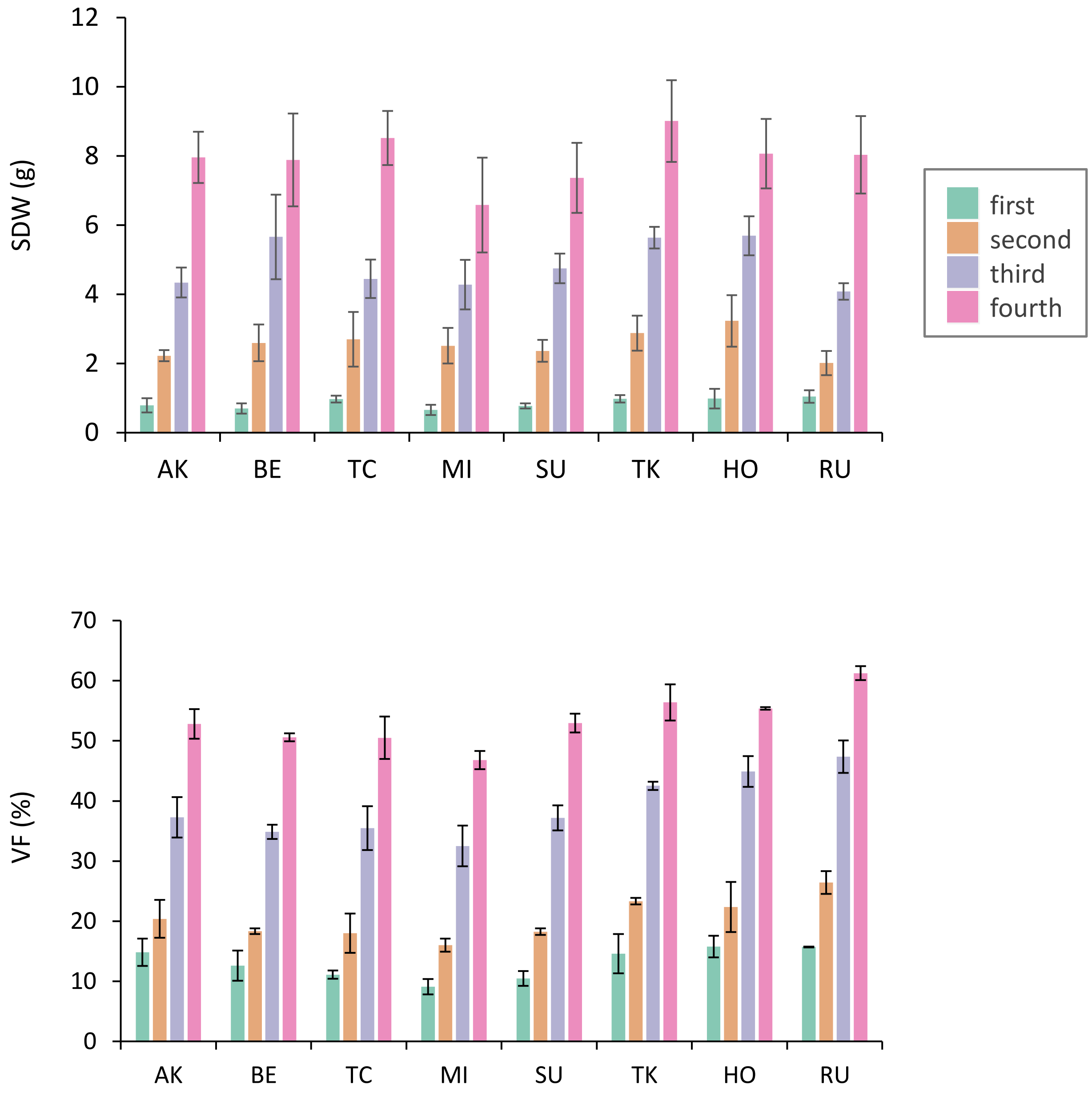

Supplementary Figure S2. Shoot dry weight and vegetation fraction of JAM's parental lines in 2019. Bars indicate mean ± standard deviation (n=3).

		2019								2018							
		SDW				VF				SDW				VF			
Year	Trait	first	second	third	fourth	first	second	third	fourth	first	second	third	fourth	first	second	third	fourth
2019	SDW_first	-	<.0001	<.0001	<.0001	<.0001	<.0001	0.0007	0.0106	<.0001	<.0001	<.0001	<.0001	0.0005	0.0003	0.0009	0.0097
2019	SDW_second	0.63	-	<.0001	<.0001	<.0001	<.0001	<.0001	<.0001	<.0001	<.0001	0.0006	0.0022	0.0009	0.0004	0.0006	0.0015
2019	SDW_third	0.49	0.66	-	<.0001	<.0001	<.0001	<.0001	<.0001	<.0001	0.0002	0.0046	0.0035	0.0004	0.0001	0.0004	0.0058
2019	SDW_fourth	0.46	0.58	0.61	-	<.0001	<.0001	<.0001	<.0001	0.0008	0.0076	0.037	0.014	0.0001	0.003	0.0031	0.0023
2019	VF_first	0.39	0.49	0.49	0.6	-	<.0001	<.0001	<.0001	0.0195	0.015	0.0967	0.0201	<.0001	<.0001	<.0001	<.0001
2019	VF_second	0.35	0.48	0.46	0.52	0.85	-	<.0001	<.0001	0.0041	<.0001	0.0011	<.0001	<.0001	<.0001	<.0001	<.0001
2019	VF_third	0.26	0.4	0.39	0.46	0.73	0.87	-	<.0001	0.0019	<.0001	0.001	<.0001	<.0001	<.0001	<.0001	<.0001
2019	VF_fourth	0.2	0.34	0.34	0.42	0.7	0.79	0.91	-	0.0466	0.0416	0.121	0.0014	<.0001	<.0001	<.0001	<.0001
2018	SDW_first	0.46	0.37	0.36	0.26	0.18	0.22	0.24	0.16	-	<.0001	<.0001	<.0001	<.0001	<.0001	<.0001	0.0004
2018	SDW_second	0.45	0.34	0.29	0.21	0.19	0.3	0.3	0.16	0.71	-	<.0001	<.0001	<.0001	<.0001	<.0001	<.0001
2018	SDW_third	0.39	0.26	0.22	0.16	0.13	0.25	0.25	0.12	0.61	0.77	-	<.0001	<.0001	<.0001	<.0001	<.0001
2018	SDW_fourth	0.35	0.24	0.23	0.19	0.18	0.31	0.37	0.25	0.48	0.69	0.73	-	<.0001	<.0001	<.0001	<.0001
2018	VF_first	0.27	0.26	0.27	0.3	0.55	0.56	0.61	0.54	0.38	0.49	0.42	0.64	-	<.0001	<.0001	<.0001
2018	VF_second	0.28	0.27	0.3	0.23	0.45	0.58	0.68	0.58	0.43	0.6	0.56	0.77	0.87	-	<.0001	<.0001
2018	VF_third	0.26	0.26	0.27	0.23	0.42	0.54	0.66	0.58	0.39	0.58	0.54	0.77	0.83	0.96	-	<.0001
2018	VF_fourth	0.2	0.25	0.21	0.24	0.42	0.53	0.7	0.65	0.27	0.45	0.43	0.69	0.78	0.9	0.93	-

Supplementary Figure S3. Pearson correlation between all shoot dry weights and vegetation fractions in two years' experiments using the JAM lines. Bottom-left and up-right show the Pearson's *r* and *P* value, respectively.

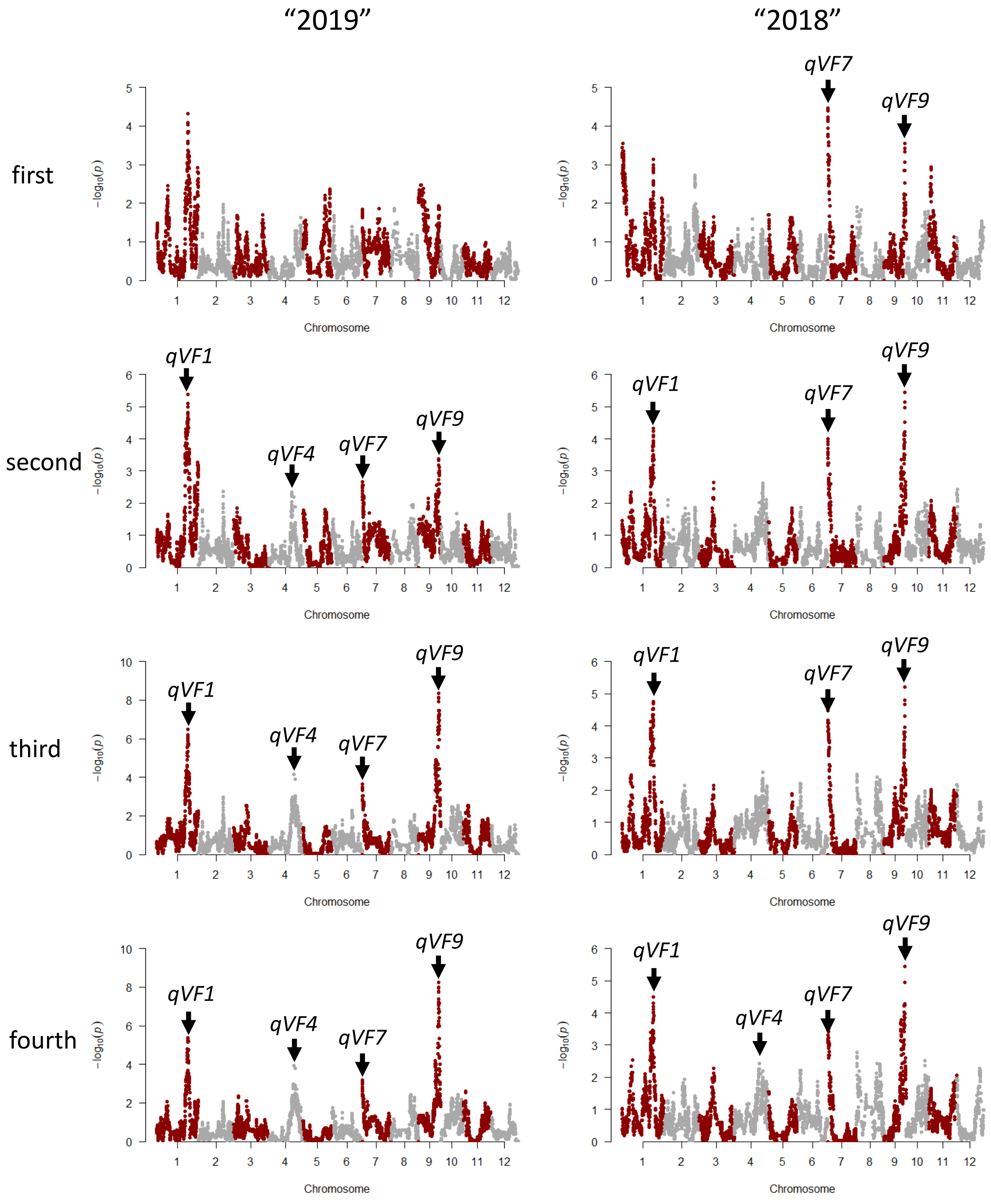

Supplementary Figure S4. Manhattan plot of GWAS regarding vegetation fraction of JAM lines in 2019 and 2018. The black arrows indicate the QTL positions of *qVF1*, *qVF4*, *qVF7* and *qVF9*.

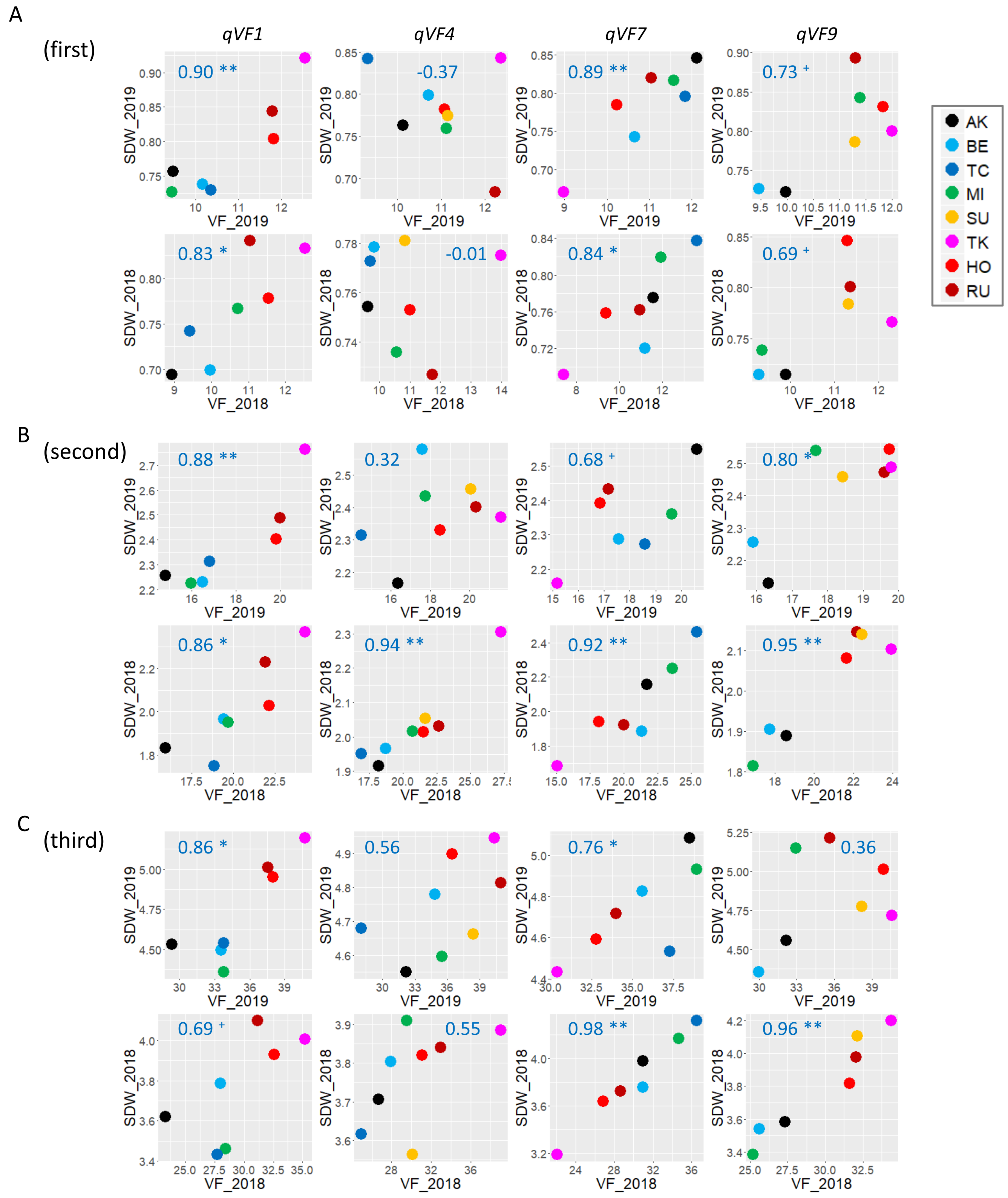

Supplementary Figure S5. Average phenotypic values of the eight haplotypes at the four QTL positions. Relationships between VF and SDW on the first (A), second (B) and third (C) sampling date in 2019 (top) and 2018 (bottom). Numbers in blue indicate Pearson's *r*. Asterisks indicate significant correlations (***P* < 0.01, **P* < 0.05, $^{+}P < 0.1$).

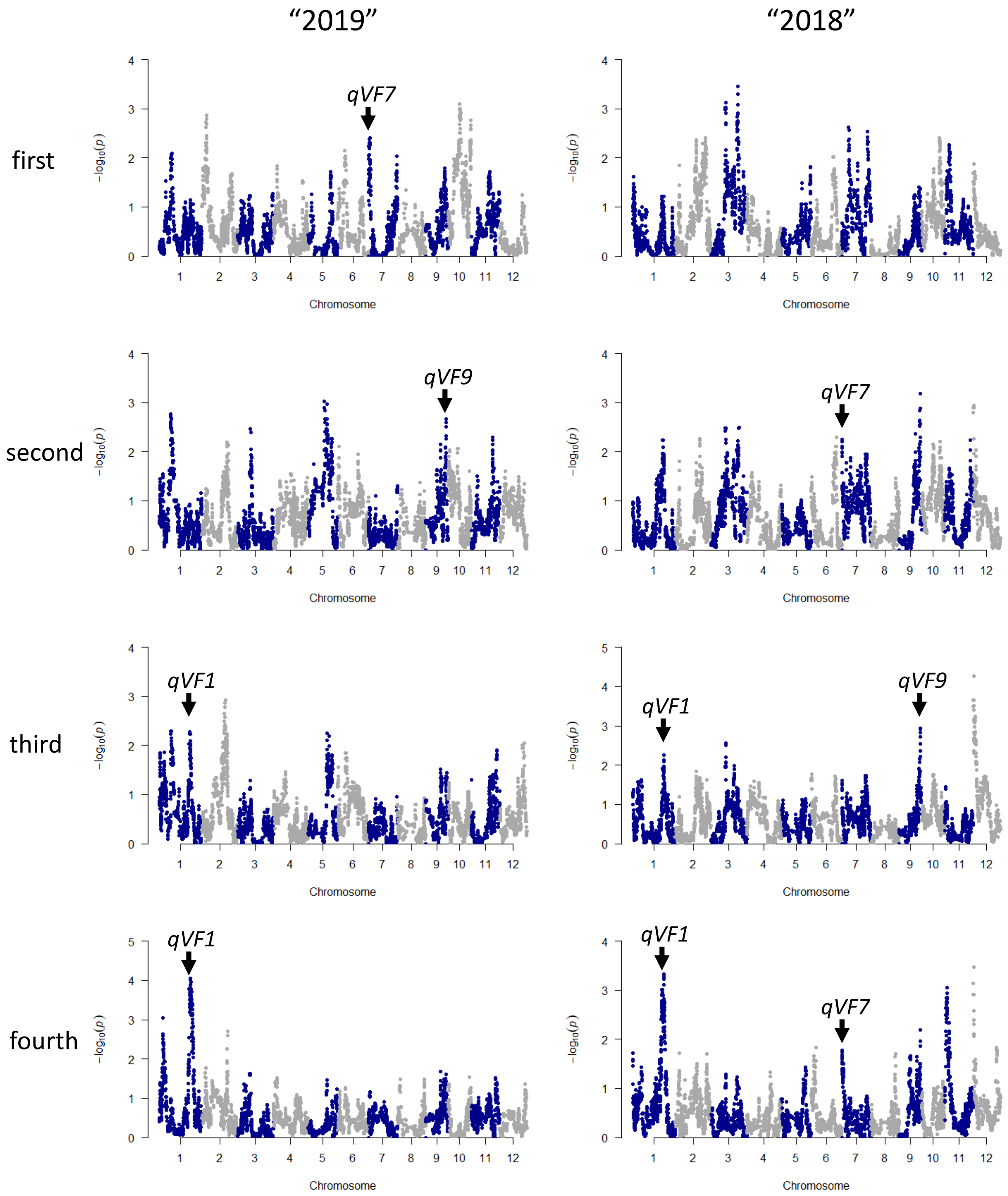

Supplementary Figure S6. Manhattan plot of GWAS regarding shoot dry weight of JAM lines in 2019 and 2018. The black arrows indicate the QTL positions of *qVF1*, *qVF4*, *qVF7* and *qVF9*.

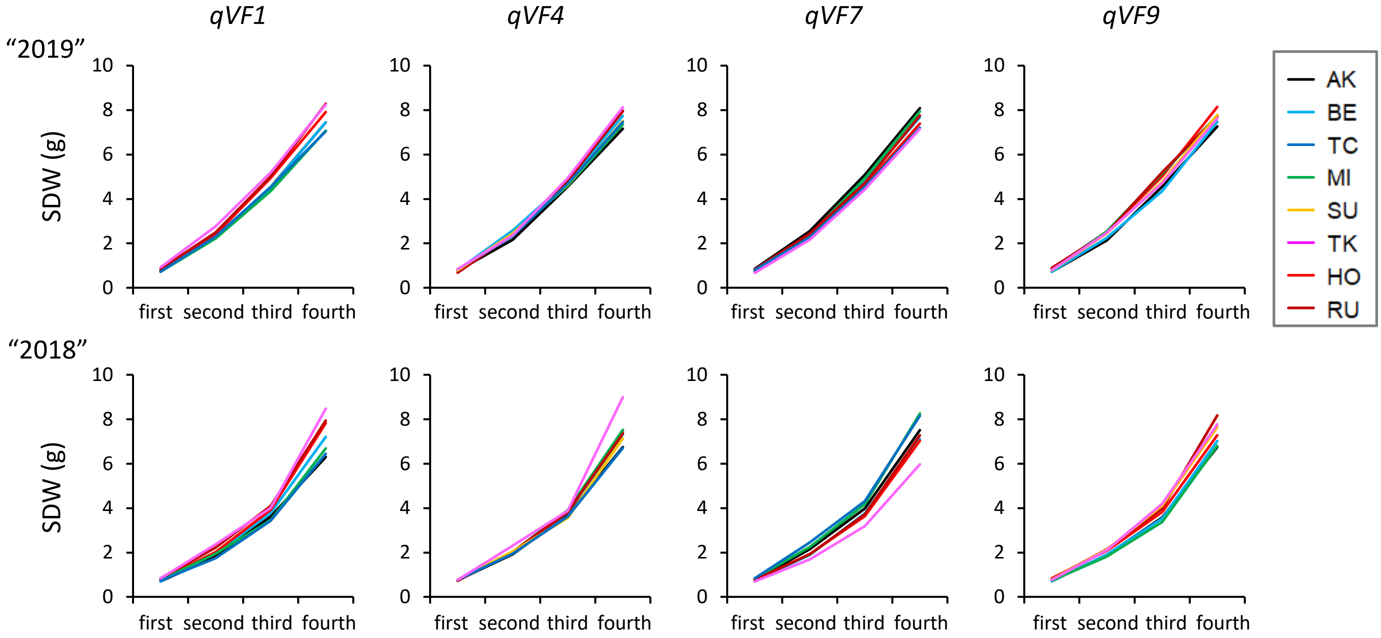

Figure S7. Time-course pattern of the effect of eight haplotypes on shoot dry weight. Average phenotypic values of the eight haplotypes at the four QTL positions from first to fourth in 2019 (top) and 2018 (bottom).

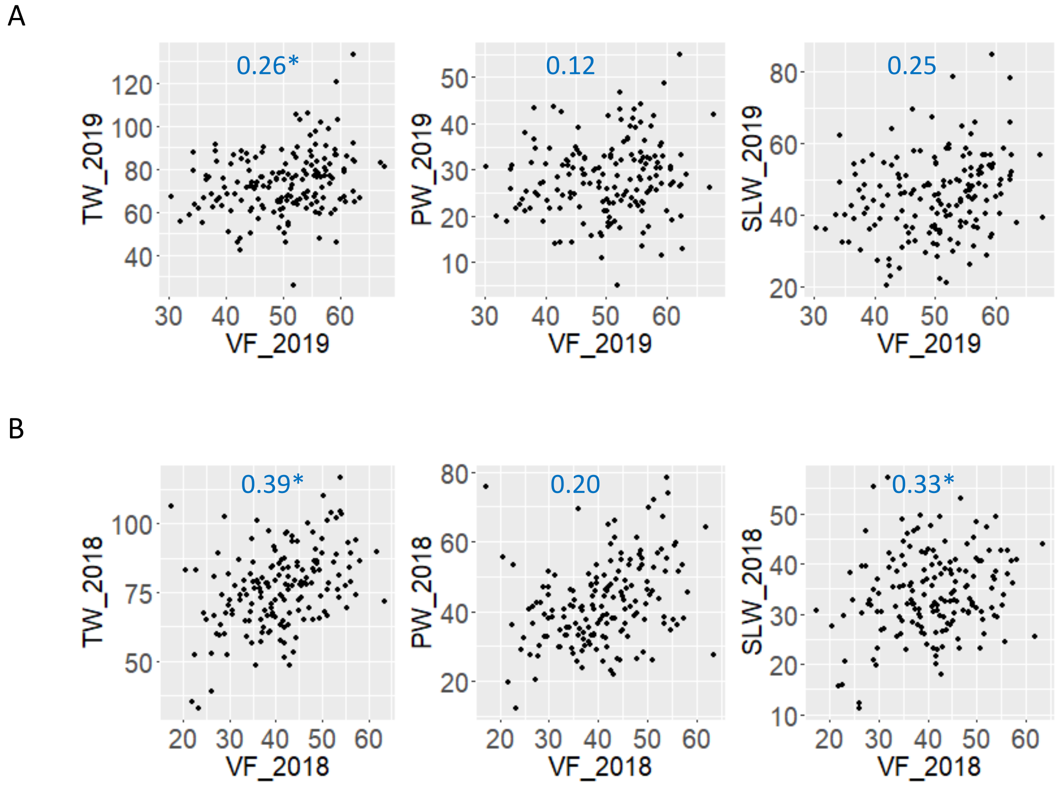

Supplementary Figure S8. Scatter plots of vegetative fraction (fourth) and yield traits. Correlation between VF on the fourth date and panicle weight (PW), stem and leaf weight (SLW), or the total weight (TW) in 2019 (A) and in 2018 (B) were shown Numbers in blue indicate Pearson's *r*. Asterisks indicate significant correlations (**P* < 0.001)

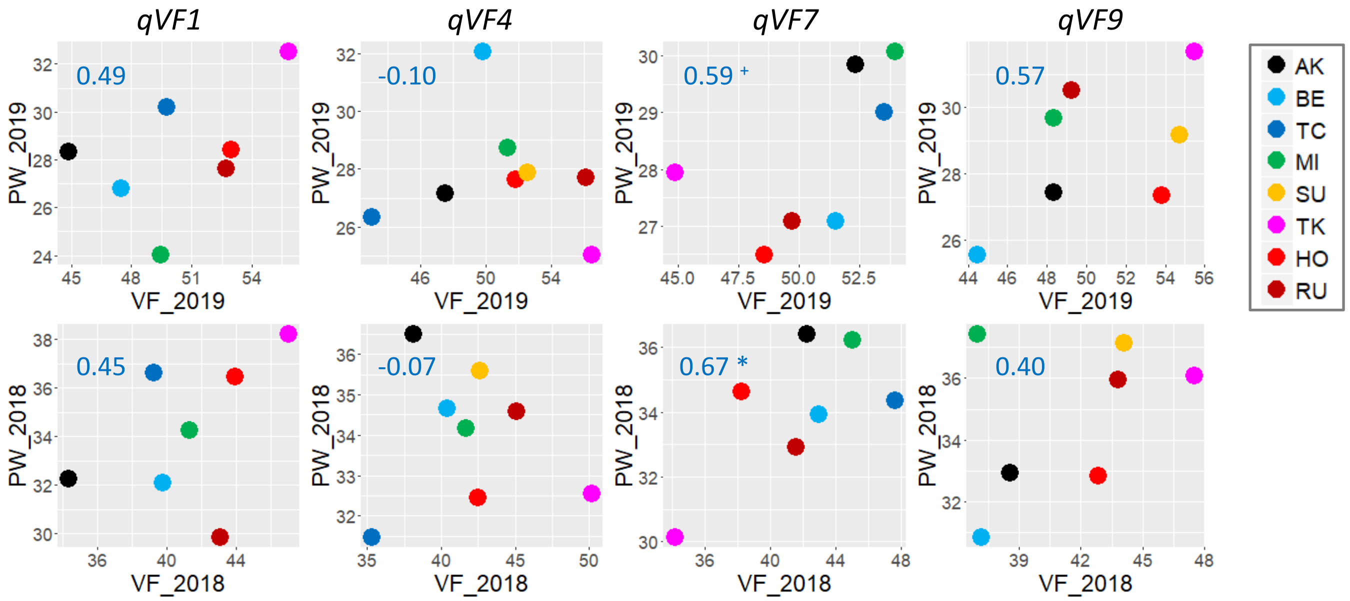

Supplementary Figure S9. Effects of the four QTLs for vegetation fractions on panicle weight. Plots of the correlation between VF and PW in 2019 (top) and 2018 (bottom) at the haplotype level. Numbers in blue indicate Pearson's r. Asterisks indicate significant correlations (*P < 0.05, ^{+}P < 0.1).

A

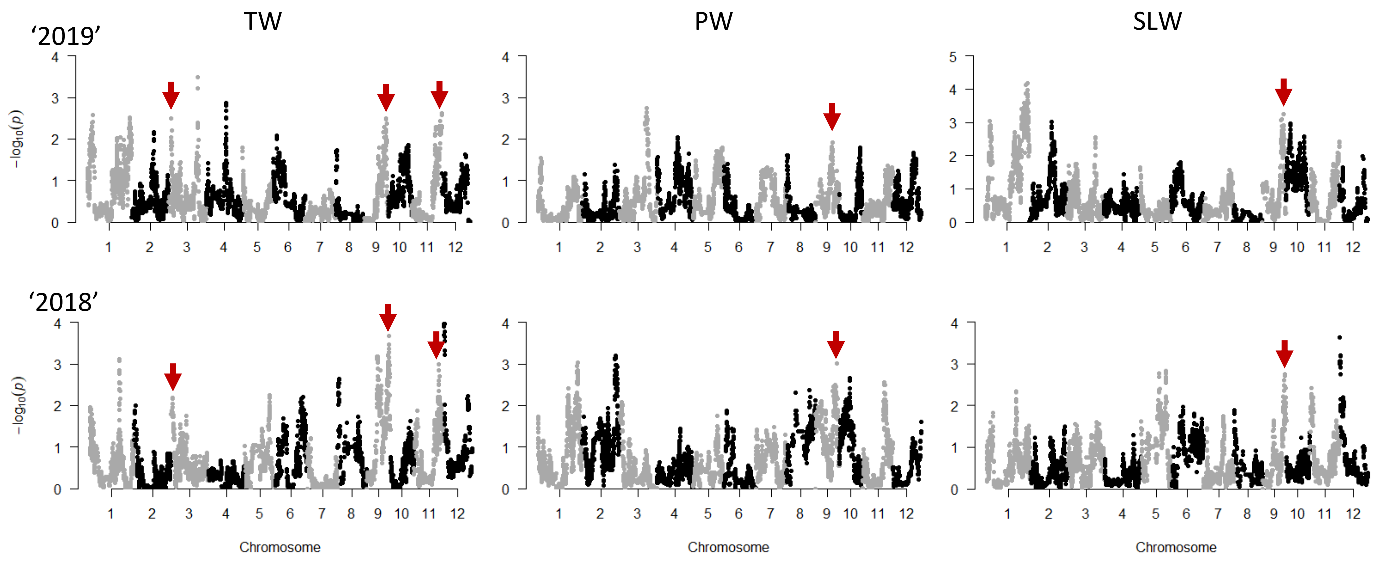

B

Phenotype	Number of QTL	*qVF1*	*qVF4*	*qVF7*	*qVF9*
TW	3	-	-	-	+
PW	1	-	-	-	-
SLW	1	-	-	-	+

Supplementary Figure S10. Haplotype-based GWAS regarding total weight, panicle weight, and stem and leaf weight. (A) Manhattan plot of GWAS regarding TW, PW and SLW of JAM lines in 2019 (top) and 2018 (bottom). Among the eight strongest QTL on each trait in 2019 and 2018, common QTL were shown in red arrows. (B) Summary of the common QTL for TW, PW and SLW. The *qVF9* was detected as QTL for TW and SLW.
